# Five-Year Changes in Physical and Cognitive Function in Individuals with Chronic Stroke: An Ambispective Cohort Study

**DOI:** 10.3390/medsci14030358

**Published:** 2026-06-30

**Authors:** Yanisa Sinthunyathum, Nantaporn Jitpimolmard, Jittima Saengsuwan

**Affiliations:** Department of Rehabilitation Medicine, Faculty of Medicine, Khon Kaen University, Khon Kaen 40002, Thailand; yanisa.sin@kkumail.com (Y.S.); nantano@kku.ac.th (N.J.)

**Keywords:** stroke, gait, cognition, chronic, predictor

## Abstract

**Background/Objectives**: This study aimed to evaluate longitudinal changes in physical and cognitive function in individuals with chronic stroke over five years and to explore factors associated with long-term outcomes. **Methods**: This ambispective cohort study included individuals with chronic stroke who had participated in a previous cross-sectional study conducted between 2018 and 2019. Assessments were performed at baseline and five-year follow-up (2023–2024). Primary outcomes were physical function, assessed using the six-minute walk test (6MWT), comfortable and fast gait speeds measured by the ten-meter walk test (10MWT), and cognitive function, assessed using the Mini-Mental State Examination (MMSE). **Results**: Thirty-two individuals participated (mean age 63.5 ± 10.3 years; median time since stroke 7.0 years). The six-minute walk distance declined by 22% (263.7 to 206.8 m, *p* < 0.001), whereas no significant changes were observed in gait speed or cognitive function. Age, baseline National Institutes of Health Stroke Scale (NIHSS) score, baseline values of the 6MWT and 10MWT, and nutritional status (Mini Nutritional Assessment–Short Form; MNA-SF) showed associations with physical outcomes. For cognitive outcomes, baseline NIHSS score, baseline MMSE score, MNA-SF score, and education level showed associations. However, sensitivity analyses suggested that the associations involving MNA-SF and education level were not robust. **Conclusions**: Physical function declines over five years in individuals with chronic stroke, highlighting the importance of long-term follow-up. While global cognition (MMSE) remained stable, domain-specific declines cannot be ruled out. Baseline stroke severity, nutritional status, and initial functional and cognitive performance may be associated with long-term outcomes.

## 1. Introduction

Stroke is a leading cause of disability worldwide, resulting in significant physical and cognitive decline during the first few years after stroke [1,2], especially among individuals with severe strokes [3]. Impairments in mobility, gait, balance, and cognition contribute to reduced quality of life and increased dependency, disability, and mortality [4]. Even stroke survivors with high premorbid functioning or minor strokes frequently experience a combination of motor and cognitive impairments [5]. Although previous studies have focused on acute and subacute phases of stroke, evidence from long-term studies, especially those focusing on physical and cognitive function in chronic stroke survivors, is limited.

Stroke survivors frequently experience physical disabilities that they perceive as a major concern. A meta-analysis of individuals with subacute stroke demonstrated significant impairment in gait performance during simple tasks, as assessed by the six-minute walk test (6MWT) and the ten-meter walk test (10MWT). The findings indicate that the average gait speed in this population is insufficient for safe street crossing and independent community ambulation [6]. Among individuals with chronic stroke, balance and walking difficulties, together with post-stroke fatigue, have been identified as the most significant challenges affecting life after stroke [7]. Mobility limitations, as measured by the Timed Up and Go (TUG) test, have been shown to be associated with age and greater stroke severity [8]. Longitudinal follow-up of previously activities-of-daily-living–independent stroke survivors revealed that approximately 3% progress to dependency each year. Although physical function may plateau during the early years after stroke, a substantial decline often occurs in later stages, underscoring that the chronic phase of stroke is not static [2,9].

Several studies have reported that a substantial proportion of patients with acute stroke experience cognitive dysfunction, as assessed by neuropsychological testing [1,10]. Such impairment markedly increases the risk of subsequent dementia, with an estimated annual incidence of approximately 3%. Individuals with lower premorbid cognitive function are particularly vulnerable to greater cognitive decline following stroke [8]. Additional independent predictors of post-stroke cognitive impairment include older age, ethnicity, lower socioeconomic status, prior stroke, left hemispheric involvement, greater stroke severity, and initial impairment deficits such as visual field defects and urinary incontinence [1,4,11]. Previous research has also identified that balance and gait impairments are significant risk markers for cognitive status after mild stroke [10]. However, most existing studies are limited to the early post-stroke period, and evidence regarding gait-related measures in individuals with chronic stroke remains scarce. Thus, this study aimed to investigate five-year changes in physical and cognitive function in individuals with chronic stroke. In addition, we aimed to generate hypotheses regarding potential factors associated with long-term outcomes after stroke.

## 2. Materials and Methods

This ambispective cohort study used longitudinal data collected as a five-year follow-up of a subset of participants originally enrolled in a cross-sectional study [12] conducted at the Rehabilitation Unit, Srinagarind Hospital, Khon Kaen University, Thailand. The study was considered ambispective because baseline data were obtained retrospectively from the original study cohort, while follow-up outcome data were collected prospectively five years later. The original study, carried out between December 2018 and October 2019, included individuals with stroke who were diagnosed at least three months prior to enrollment, were aged 18 years or older, and were able to walk a minimum distance of 10 m. All participants were community-dwelling individuals and were not institutionalized at the time of enrollment. As the present study was conducted approximately five years after the initial assessment, the shortest duration since stroke onset among participants in the current study was approximately 5 years and 3 months. The five-year follow-up assessment was conducted between November 2023 and June 2024. All patients from the previous study were targeted for recruitment. Individuals attending scheduled outpatient appointments were approached on the day of their visit and invited to participate in the follow-up assessment. Those without a scheduled appointment were contacted by mail and received an information letter with an invitation to participate. Participants who had unstable vital signs (blood pressure less than 90/60 mmHg or more than 180/100 mmHg, or heart rate less than 60 bpm or more than 140 bpm) were excluded from the study.

For sample size estimation, we considered participants from the original study who wore a waist-worn GENEA accelerometer (GENEA, GENEActiv Original, Activinsights Ltd., Kimbolton, UK), a compact tri-axial device designed to quantify movement over seven consecutive days. Participants were required to wear the accelerometer for at least 70% of the total recording period. Based on these criteria, the original dataset comprised approximately 50 participants. Allowing for an anticipated attrition and data loss rate of about 30%, the required sample size for the present study was estimated to be approximately 35 participants.

The study protocol was approved by the Khon Kaen University Ethics Committee for Human Research (Ref: HE661406) on 6 October 2023, and written informed consent was obtained from all participants.

### 2.1. Clinical Variables at Baseline and 5-Year Follow Up

Demographic characteristics, comorbidities, stroke recurrence, falls, and functional status were obtained from medical records and structured interviews. Baseline data for clinical and functional assessments—including the National Institutes of Health Stroke Scale (NIHSS), Mini-Mental State Examination (Thai Version) (MMSE), Modified Rankin Scale (mRS), Functional Ambulation Category (FAC), six-minute walk test (6MWT), and the ten-meter walk test (10MWT)—were retrieved from existing records, whereas the five-year follow-up assessments were administered in person by the research team who were blinded to the participants’ baseline clinical and functional scores. At both time points, participants completed the Mini Nutritional Assessment-Short Form (MNA-SF), Fatigue Assessment Scale [13], and Short Falls Efficacy Scale-International (Short FES-I) [14].

Physical activity was assessed using both objective and self-reported measures. Objective physical activity was evaluated exclusively at baseline using a waist-worn GENEA tri-axial accelerometer (GENEActiv Original, Activinsights Ltd., Kimbolton, UK) over seven consecutive days [12]. The specific metric analyzed was the weekly duration of moderate-to-vigorous physical activity (MVPA). For the regression analysis, this metric was dichotomized to classify participants as either active (≥150 min of MVPA per week) or non-active. In addition, self-reported physical activity was evaluated using the Stroke Physical Activity Questionnaire (SPAQ) [12,15] at both the baseline and at five-year follow-up.

For the 6MWT, participants were instructed to walk at a comfortable pace for six minutes along a level, enclosed 20-m corridor [16]. Rest periods were permitted as needed, and walking aids were allowed when required. The total distance walked during a single trial was recorded [17]. The 10MWT was used to assess both comfortable and fast gait speeds over a 10-m distance, with timing recorded for the central 6-m section. Two trials were performed for each gait speed condition, and gait speed was calculated in m per second (m/s). The mean value of the two trials was used for analysis [18,19].

### 2.2. Outcome Measurements

The primary outcomes of the study were physical and cognitive function. Physical function was evaluated using the 6MWT and the 10MWT. Cognitive function was assessed using the MMSE, with a maximum possible score of 30 points.

### 2.3. Statistical Analysis

Descriptive statistics are presented as frequencies and percentages for categorical variables, and as means with standard deviations (SDs) or medians with the 25th and 75th percentiles (P25 and P75) for continuous variables. Changes in physical and cognitive function between the baseline and five-year follow-up were analyzed using paired t-tests for parametric data and Wilcoxon signed-rank tests for non-parametric data. Changes in the proportion of participants across categories of the modified Rankin Scale (mRS) and Functional Ambulation Categories (FAC) were evaluated using McNemar’s test. There were no missing data for the baseline variables. However, 18 participants were lost to follow-up, resulting in missing outcome data at the five-year assessment. Analyses were therefore conducted using a complete-case approach, including only participants with available outcome data at both the baseline and follow-up. No imputation methods were applied.

Ten variables were examined as potential predictors in the regression analysis: sex, age, hemiparetic side, stroke type, educational level, baseline body mass index (BMI), baseline NIHSS score, baseline physical activity level, baseline MNA-SF score, and baseline value of each outcome measure. Variables demonstrating an association at *p* < 0.25 in simple linear regression analyses [20] were entered into multivariable regression models using a forward selection approach [21]. Multicollinearity was assessed using variance inflation factors (VIFs), with all predictors showing VIF values < 5 (range from 1.01 to 2.29). Model assumptions were evaluated by visual inspection of the residual histograms and Q–Q plots for normality, and heteroscedasticity was assessed using the Breusch–Pagan test. Final model selection was based on the highest adjusted coefficient of determination (adjusted R^2^), the lowest standard error of estimate (SEE), and the lowest Akaike information criterion (AIC). All statistical analyses were conducted using Stata (Stata Statistical Software: Release 18. StataCorp LLC, College Station, TX, USA).

## 3. Results

**Figure 1:**
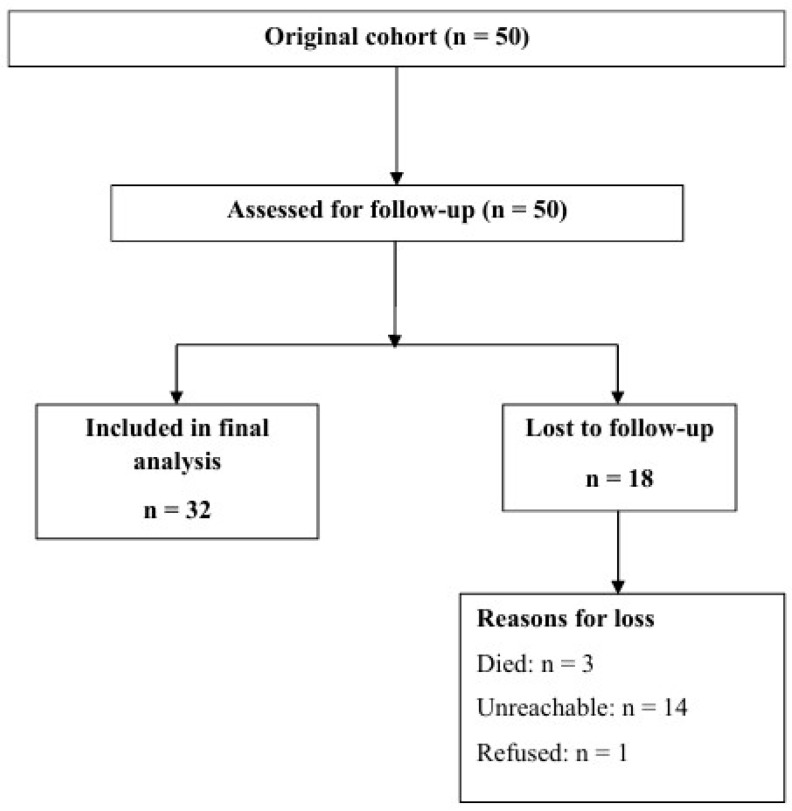
Flow diagram of the study participants.

Of the 50 patients originally enrolled in the baseline cohort, 32 successfully completed the five-year follow-up assessment. The remaining 18 participants were lost to follow-up due to death (n = 3), inability to be contacted (n = 14), or refusal to participate (n = 1). Participants were classified as unable to be contacted if they could not be reached despite repeated contact attempts, including cases with outdated contact information or no response to invitation letters (Figure 1). Baseline demographic and functional characteristics did not differ significantly between completers (n = 32) and non-completers (n = 18) (Supplementary Table S1). However, participants lost to follow-up tended to have poorer baseline functional status, as reflected by higher NIHSS scores, slower gait speeds, and shorter six-minute walk distances.

Among the analyzed cohort of 32 participants, age at the five-year follow-up was 63.5 ± 10.3 years, and 53.1% were men. The follow-up assessment was conducted at a median of 7.0 years post-stroke. Most participants had ischemic stroke (71.9%) and left-sided hemiparesis (71.9%). During this follow-up period, 3 patients (9.4%) experienced a recurrent stroke. Hypertension was the most common comorbidity, with other conditions, including chronic kidney disease, heart disease, and cancer, also reported (Table 1).

### 3.1. Physical, Cognitive and Functional Outcomes

Changes in physical function and cognition from the baseline to the five-year follow-up are summarized in Table 2. The distance covered in the 6MWT declined by 22% (263.7 to 206.8 m, *p* < 0.001). In contrast, no significant changes were observed in gait speed on the 10MWT, under either comfortable or fast walking conditions, or in cognitive function as measured by the MMSE. Additionally, no significant changes were found in stroke severity, as assessed by the National Institutes of Health Stroke Scale (NIHSS), or in the level of disability and ambulatory status, as determined by the Modified Rankin Scale (mRS) and Functional Ambulation Category (FAC).

### 3.2. Predictors of Outcomes

Factors associated with functional outcomes at the five-year follow-up are presented in Table 3. Each final multivariable model included a limited number of predictors (6MWT: 4 predictors; 10MWT comfortable speed: 4 predictors; 10MWT fast speed: 4 predictors; MMSE: 4 predictors). Baseline values of each outcome measure were significantly associated with their corresponding follow-up outcomes. Baseline NIHSS scores and MNA-SF scores were significantly associated with 6MWT, comfortable and fast gait speeds, and cognitive status. Age was significantly associated with 6MWT performance and both gait speed outcomes. Educational level was significantly associated with cognitive function, with higher education associated with better MMSE scores. Assessment of model assumptions using residuals and Q–Q plots indicated no substantial deviations from normality or evidence of heteroscedasticity. The final models explained 61% to 90% of the variance in outcomes (adjusted R^2^ range: 0.61–0.90). Sex, stroke type, hemiparetic side, recurrent stroke, and physical activity level were not significantly associated with any outcomes.

Influential observation analysis identified a small number of cases exceeding commonly used thresholds. Sensitivity analyses showed that associations involving baseline MNA-SF and educational level were more sensitive to influential observations than those involving other predictors. After the exclusion of influential cases, MNA-SF and educational level were no longer significantly associated with outcomes, whereas associations involving age, NIHSS, and baseline performance measures remained largely unchanged.

## 4. Discussion

This ambispective cohort study followed individuals with chronic stroke, with a mean age of 63.5 years and a median of 7.0 years post-stroke. Due to the rather small sample size, this study aimed to examine long-term changes in physical and cognitive function and to generate hypotheses regarding factors associated with long-term outcomes after stroke. The findings demonstrated a significant decline in physical function over time, whereas cognitive function remained relatively stable.

A decline in gait performance was observed over the five-year follow-up period, as reflected by reductions in walking endurance measured by the six-minute walk test (6MWT), while gait speed measured by the ten-meter walk test (10MWT) remained relatively unchanged. These findings are consistent with previous long-term follow-up studies. For example, Shin et al. reported that functional decline typically begins approximately 30 months after stroke onset [9]. Similarly, longitudinal studies have demonstrated a gradual transition from independence to dependency in activities of daily living over a five-year period [2], aligning with the functional changes observed in the present study. Meyer et al. reported that functional and motor performance at five years post-stroke may decline to levels comparable to those observed as early as two months after stroke [22]. 

A significant decline was observed in the 6MWT, whereas no significant change was detected in the 10MWT. This discrepancy may be attributable to the inherent characteristics and constructs of the tests. The 10MWT assesses gait performance over a very short distance, which has been shown to overestimate true locomotor capacity and may be less sensitive to functional changes related to fatigue [23]. Furthermore, in our mildly-impaired cohort, this short-distance test is likely subject to a ceiling effect, limiting its ability to detect early functional deterioration. In contrast, the 6MWT involves sustained walking over a longer duration and acts as a valid submaximal measure of cardiorespiratory endurance [24]. Mechanistically, reduced physical activity over a five-year period frequently leads to reduced cardiovascular endurance and the progression of post-stroke fatigue. When combined with the effects of natural aging and sarcopenia, these physiological factors compromise sustained walking capacity long before they substantially impact short-burst gait speed. Consequently, the 6MWT may be more sensitive to cumulative global functional changes, allowing performance differences to become more apparent. Although the observed decline was statistically significant, the magnitude of change did not exceed the minimal clinically important difference reported by Fulk et al. [25]. Therefore, the observed decline may have limited clinical relevance despite its statistical significance.

Physical function at follow-up, as assessed by the 6MWT and both comfortable and fast gait speeds, was significantly associated with age, NIHSS scores, MNA-SF scores, and baseline performance on each corresponding test. These findings suggest that physical function in the post-stroke period demonstrated associations with long-term functional outcomes. Previous studies have demonstrated that improvements in nutritional status during hospitalization can enhance functional outcomes, as measured by the Functional Independence Measure (FIM), and facilitate more efficient recovery of activities of daily living [26,27,28]. Consistent with this evidence, the present study indicates that poorer baseline nutritional status may be associated with worse long-term physical function in individuals with chronic stroke. In contrast to previous reports highlighting the protective role of physical activity, neither baseline accelerometer-derived activity status (active vs. non-active) nor self-reported physical activity (SPAQ) was significantly associated with long-term functional outcomes in the current study. This lack of significance may be attributable to the relatively small sample size, which limited the statistical power. Furthermore, the homogeneity of the predominantly mildly impaired and ambulatory cohort might have restricted the variance required to detect meaningful associations. Finally, the dichotomization of physical activity data and the reliance on a single seven-day baseline assessment may not adequately capture dynamic lifestyle changes over the five-year follow-up period.

Cognitive function, as assessed by the MMSE, did not show a significant decline over the five-year follow-up period. This finding aligns with previous research suggesting that stroke survivors may experience a period of relative global cognitive stability lasting up to eight years post-stroke, prior to the onset of potential decline [1], though this apparent stability is limited by the measurement tool. Stroke severity, baseline cognitive performance, and nutritional status emerged as potential factors associated with long-term cognitive outcomes. Previous studies in acute stroke populations indicated that patients with improved nutritional status demonstrated greater gains in cognitive function, as measured by the cognitive component of the Functional Independence Measure (FIM), compared with those who remained malnourished [26,27]. Similarly, longitudinal studies in community-dwelling older adults suggest that poorer nutritional status adversely affects cognitive function in later life [28], with nutritional biomarkers identified as independent predictors of subsequent cognitive decline [29]. Consistent with these findings, our results suggest nutritional status may be an important factor associated with long-term outcomes, underscoring the potential value of early identification and management of malnutrition in individuals with chronic stroke to support both functional and cognitive recovery.

A major strength of this study is its longitudinal design, which followed individuals with chronic stroke over a five-year period, allowing for a comprehensive assessment of long-term physical and cognitive outcomes. Nutritional status was identified as a potential factor associated with long-term functional and cognitive outcomes, highlighting its possible role as a modifiable target in post-stroke care. However, given the exploratory nature of the analyses and the sensitivity of this association to influential observations, this finding should be interpreted with caution. As a preliminary investigation, this study provides valuable insights that may inform future larger-scale studies and support the development of targeted interventions aimed at optimizing long-term outcomes after stroke. The factors identified in this study should be regarded as hypothesis-generating and require confirmation in independent cohorts.

Several limitations should be acknowledged. First, the long interval between the baseline assessment and the five-year follow-up prevented a detailed characterization of recovery trajectories or functional deterioration occurring at intermediate time points. Attrition occurred because of death, relocation, inability to contact, or refusal to participate, which may lead to selection bias and limit the generalizability of the findings. 

Second, a substantial proportion of participants (36 %) was lost to follow-up, which represents an important limitation and may have introduced selection bias. Although no significant differences were observed in the baseline demographic or functional characteristics between participants who completed and did not complete the follow-up assessment, those lost to follow-up tended to have poorer baseline function, as reflected by higher NIHSS scores, slower gait speed, and shorter six-minute walk distance. Given the limited sample size, these differences may not have been detected statistically. Therefore, the missing data mechanism cannot be assumed to be completely random, and attrition bias cannot be excluded. Consequently, the observed changes in physical and cognitive function may not fully represent those of the original cohort. In particular, mobility-related outcomes may have been biased toward participants with better functional status who remained in the study, potentially leading to an underestimation of functional decline in individuals with chronic stroke. 

Third, we were unable to track changes in participants’ ongoing rehabilitation, exercise habits, medication use, recurrent vascular events and their management, or the effects of COVID-19-related restrictions over the five-year follow-up period. As these factors may substantially influence recovery trajectories, our findings likely reflect the combined effects of the natural course of stroke and these unmeasured lifestyle and medical changes, rather than the isolated effects of baseline predictors alone. Furthermore, physical activity was measured objectively using accelerometry only at the baseline and used to classify participants as meeting or not meeting the World Health Organization recommendation for MVPA [30]. Although physical activity was reassessed during follow-up using questionnaire data, objective measures were not repeated. Consequently, changes in objectively measured physical activity over time and the persistence of baseline activity classification during follow-up could not be determined. In addition, MVPA was analyzed as a dichotomous variable, and some information may have been lost, thus potentially limiting our ability to detect dose–response relationships between physical activity and long-term outcomes.

Fourth, the relatively small sample size limits statistical power and may have reduced the ability to detect potentially meaningful associations. Accordingly, these findings should be considered hypothesis-generating, and the proposed prediction equations should be regarded as preliminary. Because the models were developed and evaluated within the same dataset without internal (e.g., cross-validation) or external validation, their predictive performance may be overestimated. In addition, the limited sample size and the use of forward selection may have increased the risk of overfitting and unstable regression estimates.

Although forward selection was used to derive more parsimonious models, this approach is sensitive to sample variation and may contribute to model instability. Sensitivity analyses indicated that the associations involving baseline MNA-SF score and education level were less robust than those for other factors. After the exclusion of influential observations, baseline MNA-SF was no longer significantly associated with the outcomes, whereas age, NIHSS score, and baseline performance measures remained consistent predictors. These findings suggest that the observed associations involving nutritional status should be interpreted with caution and require confirmation in larger independent cohorts.

Alternative approaches such as penalized regression techniques (e.g., LASSO or ridge regression) may provide more stable estimates and should be considered in future studies with larger samples. Overall, external validation is required before these equations can be applied in clinical settings.

Fifth, the study sample consisted predominantly of community-dwelling individuals who were independent in activities of daily living and ambulatory, reflecting mild to moderate stroke-related disability. As a result, the findings may not be generalizable to individuals with more severe impairments or those living in institutional settings. 

Sixth, a limitation of this study is the reliance on the MMSE for cognitive assessment. Because this was an ambispective study building upon a baseline cohort, we were constrained to use the MMSE to ensure valid longitudinal comparisons. However, the MMSE alone may not provide a comprehensive assessment of post-stroke cognition because it has limited sensitivity for detecting impairments in executive function, processing speed, attention, and visuospatial abilities, domains that are commonly affected after stroke. Additionally, because the cohort was relatively mildly impaired, the MMSE may have been subject to ceiling effects, potentially limiting its ability to detect subtle cognitive changes over time. Therefore, although global cognitive scores on the MMSE did not change significantly over the five-year follow-up period, changes in specific cognitive domains cannot be excluded and may have remained undetected. Furthermore, the absence of an age-matched control group limits interpretation, as the observed changes likely reflect a combination of age-related and stroke-specific effects. 

Future studies with larger sample sizes and shorter assessment intervals are warranted. Expanding follow-up modalities (e.g., motor and sensory outcomes) and incorporating domain-specific cognitive assessments may help detect abnormalities not identified by the MMSE. Longer follow-up durations and inclusion of a healthy control group are also recommended to better characterize long-term functional trajectories after stroke.

## 5. Conclusions

Individuals with chronic stroke experience long-term declines in physical function, underscoring the importance of sustained follow-up beyond the early post-stroke period. Global cognitive scores, as measured by the MMSE, showed no significant decline; however, given the test’s limited sensitivity, subtle or domain-specific cognitive decline cannot be excluded. Baseline stroke severity, nutritional status, and initial functional and cognitive performance may be associated with long-term outcomes and could represent potential targets for intervention; however, these findings require confirmation in larger studies. Importantly, sensitivity analyses revealed that the associations involving nutritional status and education level were not robust, emphasizing the need for cautious interpretation. These factors may represent potential targets for intervention. Our findings, based on a predominantly community-dwelling cohort with mild-to-moderate disability, support the consideration of nutritional assessment and long-term rehabilitation strategies in chronic stroke care. Given the exploratory nature of the analyses and the relatively small sample size, the identified factors should be considered hypothesis-generating and require confirmation in larger independent cohorts. Further studies with larger sample sizes and more frequent follow-up assessments are warranted to validate these findings and further elucidate the roles of physical activity and nutrition in long-term stroke recovery.

## Figures and Tables

**Table 1:** Demographic and clinical characteristics of participants at the 5-year follow-up (n = 32).

Variables	n (%)
**Age** (years), mean ± SD	63.5 ± 10.3
**Sex**	
Male, n (%)	17 (53.1)
Female, n (%)	15 (46.9)
Current smoking, n (%)	1 (3.1)
**Marital status, n (%)**	
Married	25 (78.1)
Single/Separated/Divorced	7 (21.9)
**Educational level, n (%)**	
≤secondary school	16 (50.0)
>secondary school	16 (50.0)
**Body mass** (kg), mean ± SD	66.9 ± 11.9
**Height** (cm), mean ± SD	161.3 ± 6.6
**BMI** (kg/m^2^), mean ± SD	25.6 ± 3.8
**Comorbidities, n (%)**	
Hypertension	21 (65.6)
Diabetes mellitus	11 (34.4)
Dyslipidemia	19 (59.4)
Chronic kidney disease	3 (9.4)
**Stroke duration** (years), median (P25, P75)	7.0 (6.1, 10.6)
**Stroke type, n (%)**	
Infarction	23 (71.9)
Hemorrhage	9 (28.1)
**Hemiparetic side, n (%)**	
Right	9 (28.1)
Left	23 (71.9)
**Recurrent stroke**	3 (9.4)

Abbreviations: SD, standard deviation; BMI, body mass index; P25, 25th percentile; P75, 75th percentile.

**Table 2:** Changes in clinical and functional outcomes between the baseline and 5-year follow-up (n = 32).

Variables	Baseline	5-Year Follow-Up	Mean Difference (95% CI)	*p*-Value
NIHSS score, median (P25, P75)	3 (2, 5)	3 (1, 4)	–	0.99
mRS, n (%)				
0–2	25 (78.1)	25 (78.1)	–	1.00
3–4	7 (21.9)	7 (21.9)		
FAC, n (%)				
0–3	1 (3.1)	3 (9.4)	–	1.00
4–5	31 (96.9)	29 (90.6)		
Six-minute walk distance (m), mean ± SD	263.7 ± 151.3	206.8 ± 139.8	−56.8 (−88.1 to −25.6)	<0.001
Comfortable gait speed (m/s), mean ± SD	0.69 ± 0.39	0.66 ± 0.45	−0.03 (−0.11 to 0.05)	0.49
Fast gait speed (m/s), mean ± SD	0.85 ± 0.49	0.80 ± 0.54	−0.05 (−0.15 to 0.05)	0.33
MMSE score, median (P25, P75)	25.5 (24.0, 29.0)	26 (24.0, 28.0)	–	0.10
Barthel index, median (P25, P75)	100.0 (95.0, 100.0)	100.0 (92.5, 100.0)	–	0.62
MNA-SF score, median (P25, P75)	12 (10, 13)	12 (10, 13)	–	0.68
MNA-SF categories, n (%)				
12–14 points	17 (53.1)	18 (56.2)	–	0.85
8–11 points	13 (40.6)	12 (37.5)		
0–7 points	2 (6.2)	2 (6.2)		
Short Falls Efficacy Scale, median (P25, P75)	12.0 (7.0, 14.5)	10.0 (7.0, 14.0)	–	0.48
Global quality of life, median (P25, P75)	80.0 (65.0, 87.5)	75.0 (60.0, 82.5)	–	0.39

Abbreviations: CI, confidence interval; FAC, Functional Ambulation Category; NIHSS, National Institutes of Health Stroke Scale; MMSE, Mini-Mental State Examination; mRS, Modified Rankin Scale; MNA-SF, Mini Nutritional Assessment–Short Form; P25, 25th percentile; P75, 75th percentile.

**Table 3:** Univariate and multivariable linear regression analyses of factors associated with physical and cognitive outcomes at the 5-year follow-up.

Factors	Univariate β (95% CI)	*p*-Value	Multivariable β (95% CI)	*p*-Value
**6-min walk distance (m)**				
Age (y)	−4.59 (−9.16 to −0.01)	0.050	−3.35 (−5.46 to −1.23)	0.002
Baseline NIHSS score	−28.00 (−38.40 to −17.59)	<0.001	−11.70 (−20.29 to −3.11)	0.008
Baseline 6-min walk distance (m)	0.76 (0.58 to 0.95)	<0.001	0.52 (0.33 to 0.72)	<0.001
Baseline MNA-SF score	24.54 (4.88 to 44.19)	0.014	14.82 (5.69 to 23.95)	0.001
Constant	NA	NA	155.72 (−61.94 to 373.38)	0.161
**Comfortable gait speed (m/s)**				
Age (y)	−0.02 (−0.03 to −0.01)	0.009	−0.02 (−0.02 to −0.01)	<0.001
Baseline NIHSS score	−0.09 (−0.12 to −0.06)	<0.001	−0.04 (−0.06 to −0.02)	<0.001
Baseline comfortable gait speed (m/s)	0.98 (0.76 to 1.20)	<0.001	0.65 (0.46 to 0.85)	<0.001
Baseline MNA-SF score	0.07 (0.01 to 0.13)	0.035	0.03 (0.01 to 0.05)	0.009
Constant	NA	NA	0.95 (0.39 to 1.52)	0.001
**Fast gait speed (m/s)**				
Age (y)	−0.02 (−0.04 to −0.00)	0.014	−0.02 (−0.03 to −0.01)	<0.001
Baseline NIHSS score	−0.10 (−0.14 to −0.07)	<0.001	−0.05 (−0.08 to −0.02)	0.001
Baseline fast gait speed (m/s)	0.94 (0.74 to 1.15)	<0.001	0.63 (0.40 to 0.85)	<0.001
Baseline MNA-SF score	0.08 (−0.00 to 0.16)	0.047	0.04 (0.01 to 0.07)	0.016
Constant	NA	NA	1.09 (0.36 to 1.82)	0.003
**MMSE score**				
Baseline NIHSS score	−1.46 (−2.13 to −0.79)	<0.001	−1.02 (−1.71 to −0.33)	0.004
Baseline MMSE (score)	0.61 (0.24 to 0.99)	0.001	0.36 (0.01 to 0.71)	0.045
Baseline MNA-SF score	1.24 (0.05 to 2.43)	0.041	1.17 (0.31 to 2.02)	0.007
High educational level (vs. low)	4.56 (−1.01 to 10.14)	0.11	5.02 (1.20 to 8.84)	0.010
Constant	NA	NA	2.56 (−12.91 to 18.04)	0.75

Abbreviations: β, unstandardized regression coefficient; CI, confidence interval; MMSE, Mini-Mental State Examination; MNA-SF, Mini Nutritional Assessment–Short Form; NIHSS, National Institutes of Health Stroke Scale; NA, not applicable.

## Data Availability

The original contributions presented in this study are included in the article/Supplementary Materials. Further inquiries can be directed to the corresponding author.

## Supplementary Materials

The following supporting information can be downloaded at: https://www.mdpi.com/article/10.3390/medsci14030358/s1, Table S1: Baseline characteristics of participants who completed and did not complete the 5-year follow-up assessment.

## Abbreviations

The following abbreviations are used in this manuscript:

10MWT	Ten-meter walk test
6MWT	Six-minute walk test
AIC	Akaike information criterion
BMI	Body mass index
CI	Confidence interval
FAC	Functional Ambulation Category
MMSE	Mini-Mental State Examination
MNA-SF	Mini Nutritional Assessment-Short Form
mRS	Modified Rankin Scale
NA	Not applicable
NIHSS	National Institutes of Health Stroke Scale
P25	25th percentile
P75	75th percentile
SD	Standard deviation
SEE	Standard error of estimate
