# Supplementary material for: Five-Year Changes in Physical and Cognitive Function in Individuals with Chronic Stroke: An Ambispective Cohort Study
Source: Med Sci (Basel). 2026 Jun 30;14(3):358. doi: 10.3390/medsci14030358 (PMC13413838; doi:10.3390/medsci14030358)
Supplement: Supplementary file 1 [file medsci-14-00358-s001.zip › medsci-4351666-supplementary.pdf]

Supplementary Table S1 Baseline characteristics of participants who completed and did not complete the 5-year follow-up assessment

**Variables**	**Completers (n = 32)**	**Non-completers (n = 18)**	**p-value**
Age (years), mean ± SD	58.4 ± 10.1	55.2 ± 11.2	0.30
Male, n (%)	19 (59.4)	14 (77.8)	0.23
**Current smoking, n (%)**	0	1 (5.6)	0.36
BMI (kg/m^2), mean ± SD	25.0 ± 4.1	24.3 ± 3.4	0.55
**Comorbidities, n (%)**			
Hypertension	14 (43.8)	10 (55.6)	0.42
Diabetes mellitus	8 (25.0)	7 (38.9)	0.35
Dyslipidemia	5 (15.6)	2 (11.1)	1.00
**Stroke type, n (%)**			
Infarction	23 (71.9)	13 (72.2)	0.98
Haemorrhage	9 (28.1)	5 (27.8)	
**Hemiparetic side, n (%)**			
Right	9 (28.1)	5 (27.8)	0.98
Left	23 (71.9)	13 (72.2)	
**Baseline function**			
NIHSS total score, median (P25, P75)	3 (2, 5)	5 (3, 6)	0.08
Comfortable gait speed (m/s), mean ± SD	0.69 (0.39)	0.53 (0.29)	0.13
Six-minute walk distance (m), mean ± SD	263.7 ± 151.3	191.8 ± 111.6	0.11
MMSE (0-30), median (P25-P75)	25.5 (24-29)	25 (23-27)	0.28

Abbreviations: BMI, body mass index; MMSE, Mini-Mental State Examination; NIHSS, National Institutes of Health Stroke Scale; P25, 25th percentile; P75, 75th percentile P25, 25th percentile; P75, 75th percentile; SD, standard deviation
